# Real-World Effectiveness, Safety, and Health-Related Quality of Life in Patients Receiving Adjuvant Nivolumab for Melanoma in Belgium and Luxembourg: Results of PRESERV MEL †

**DOI:** 10.3390/cancers15194823

**Published:** 2023-09-30

**Authors:** Anne Rogiers, Laurence Willemot, Laura McDonald, Hilde Van Campenhout, Guy Berchem, Celine Jacobs, Nathalie Blockx, Andrée Rorive, Bart Neyns

**Affiliations:** 1 Departement of Psychiatry, Centre Hospitalier Universitaire Brugmann, 1020 Brussels, Belgium; 2 Department of Medical Oncology, Universitair Ziekenhuis Brussel, 1090 Brussels, Belgium; 3 Faculty of Medicine and Pharmacy Vrije Universiteit Brussel, 1050 Brussels, Belgium; 4 Bristol Myers Squibb, 1420 Braine L’Alleud, Belgium; 5 Bristol Myers Squibb, Uxbridge UB8 1DH, UK; 6 Centre Hospitalier de Luxembourg, University of Luxembourg, 1210 Luxembourg, Luxembourg; 7 Medical Oncology, Universitair Ziekenhuis Gent, 9000 Gent, Belgium; 8 Ziekenhuis Netwerk Antwerpen Middelheim, 2020 Antwerp, Belgium; 9 Centre Hospitalier Universitaire de Liège Sart-Tilman, 4000 Liege, Belgium

**Keywords:** adjuvant treatment, effectiveness, health-related quality of life, melanoma, nivolumab, real-world, safety

## Abstract

**Simple Summary:**

PRESERV MEL (Prospective and REtrospective Study of nivolumab thERapy in adjuVant MELanoma) is a real-world observational study evaluating the effectiveness and safety of adjuvant nivolumab in patients with completely resected stage III or stage IV melanoma in clinical practice in Belgium and Luxembourg. Patients received nivolumab for up to 12 months per label. The study enrolled 152 patients. At a minimum potential follow-up of 11.4 months, the 12-month and 18-month relapse-free survival rates were 74.7% and 68.4%, respectively, and median relapse-free survival was not reached. Grade 3 or 4 treatment-related adverse events were reported in 14% of patients. Cancer-specific, disease-specific, and generic health-related quality of life were maintained during and after treatment. These results confirm the real-world effectiveness and safety of nivolumab as an adjuvant treatment for patients with completely resected stage III or stage IV melanoma.

**Abstract:**

Background: Nivolumab, an anti–programmed cell death 1 immuno-oncology therapy, is approved as an adjuvant treatment for patients with completely resected stage III or stage IV melanoma. PRESERV MEL (Prospective and REtrospective Study of nivolumab thERapy in adjuVant MELanoma) is a real-world observational study evaluating the effectiveness and safety of adjuvant nivolumab in patients with completely resected stage III or stage IV melanoma in clinical practice in Belgium and Luxembourg. Methods: Patients were enrolled prospectively and retrospectively during a 2-year period (January 2019–January 2021), and will be followed for 5 years. The results reported here are for the second interim analysis (cutoff date 31 December 2021). The index date was the date of first administration of adjuvant nivolumab. Patients received nivolumab for up to 12 months per label. Outcomes included relapse-free survival (RFS), adverse events (AEs)/treatment-related AEs (TRAEs), and health-related quality of life (HRQoL; assessed in prospectively enrolled patients using the European Organisation for Research and Treatment of Cancer Quality of Life Questionnaire C30 (EORTC QLQ-C30), Functional Assessment of Cancer Therapy—Melanoma (FACT-M), and EQ-5D-3L instruments). HRQoL was evaluated at group level (mean change in scores from baseline based on minimally important differences) and individual patient level (percentage of patients with clinically important scores based on threshold of clinical importance). Outcomes were analyzed descriptively. Results: The study enrolled 152 patients (125 prospective, 27 retrospective) at 15 hospitals in Belgium and Luxembourg. Minimum potential follow-up at time of analysis was 11.4 months. Median age was 60 years (range 29–85), and 53% of patients were male. At 12 and 18 months, the RFS rates were 74.7% (95% confidence interval (CI): 66.9–80.9) and 68.4% (95% CI: 60.0–75.5), respectively. Median RFS was not reached. Grade 3 or 4 TRAEs were reported in 14% of patients. AEs led to treatment discontinuation in 23% of patients. Deaths occurred in 3% of patients and were not related to treatment. Questionnaire completion rates for HRQoL were high at baseline (90–94%) and at 24 months (78–81%). In the group-level analysis for HRQoL, mean changes in scores from baseline remained stable and did not exceed prespecified thresholds for minimally important differences during and after treatment, except for a clinically meaningful improvement in FACT-M surgery subscale scores. In the individual patient-level analysis for EORTC QLQ-C30 subscales, the percentages of patients who reported clinically relevant scores for fatigue and cognitive impairment increased during treatment (at 9 months) compared with baseline. After treatment cessation (at 18 months), the percentage of patients who reported clinically relevant scores for fatigue decreased. However, the percentages of patients who reported clinically relevant scores for emotional, cognitive, and social impairment increased at 18 months compared with during treatment. Most patients with emotional impairment at 9 and 18 months did not experience disease recurrence (91% and 89%, respectively). Conclusions: These results confirm the real-world effectiveness and safety of nivolumab as an adjuvant treatment for patients with completely resected stage III or stage IV melanoma. Cancer-specific, disease-specific, and generic HRQoL were maintained during and after treatment. The percentage of patients reporting emotional and cognitive impairment increased after treatment cessation, emphasizing the need for further investigation and tailored supportive care in these patients.

## 1. Introduction

Nivolumab, an anti–programmed cell death 1 (PD-1) immuno-oncology (I-O) therapy, is approved and regarded as a standard adjuvant treatment option for patients with completely resected stage III or stage IV melanoma based on findings of the pivotal phase III CheckMate 238 trial [1]. Patients in CheckMate 238 were randomly assigned to treatment with adjuvant nivolumab or ipilimumab, an anti–cytotoxic T lymphocyte antigen 4 I-O therapy, after complete resection of stage IIIB/C or stage IV melanoma (as per the *AJCC Cancer Staging Manual*, seventh edition (AJCC-7)) for a maximum of 1 year [1]. Among patients treated with adjuvant nivolumab in CheckMate 238, the 12-month relapse-free survival (RFS) rate was 70.5% (95% confidence interval (CI): 66.1–74.5) at a minimum follow-up of 18 months [1]. Grade 3 or 4 treatment-related adverse events (TRAEs) were reported in 14.4% of patients, treatment discontinuation due to any adverse event (AE) occurred in 9.7% of patients, and health-related quality of life (HRQoL) was stable compared with baseline [1]. An updated analysis of CheckMate 238 demonstrated a 48-month RFS rate of 51.7% (95% CI: 46.8–56.3) among patients treated with adjuvant nivolumab, indicating long-term benefit of treatment [2]. In the updated analysis of CheckMate 238, patients who received adjuvant nivolumab showed stable HRQoL during treatment and long-term follow-up [3]. In the phase III CheckMate 915 trial, which compared adjuvant therapy with nivolumab plus ipilimumab and nivolumab alone in patients with resected stage IIIB–D or IV melanoma (as per the AJCC *Cancer Staging Manual*, eighth edition (AJCC-8)), adjuvant nivolumab demonstrated a 24-month RFS rate of 63.2% (minimum follow-up 23.7 months) [4]. Based on the primary analysis of CheckMate 238 [1], nivolumab was approved in 2018 by the European Medicines Agency as an adjuvant treatment for patients with resected melanoma with involvement of lymph nodes or metastatic disease who have undergone complete resection [5], and it became the first anti–PD-1 therapy to be reimbursed in Belgium and Luxembourg as an adjuvant melanoma treatment.

Data from real-world studies may complement those of randomized controlled trials (RCTs) by helping to determine whether RCT results are generalizable to routine clinical practice [6]. RCTs have stringent enrollment criteria and thus may not reflect patients in routine clinical practice [6]. PRESERV MEL (Prospective and REtrospective Study of nivolumab thERapy in adjuVant MELanoma) is a real-world, observational, multicenter study evaluating the effectiveness and safety of adjuvant nivolumab in patients with completely resected stage III or stage IV melanoma (as per AJCC-8) in clinical practice in Belgium and Luxembourg [7,8]. Preliminary results of PRESERV MEL have been reported (median follow-up was 9.2 months (range 0–26)) [7]. Updated results with longer follow-up from the second interim analysis are presented here. In addition to effectiveness and safety, HRQoL was assessed in PRESERV MEL using patient-reported outcomes to better understand the impact of adjuvant nivolumab on patient well-being in a real-world setting [8].

Patient-reported HRQoL is widely accepted as a multidimensional concept that reflects how disease and treatment affect a patient’s perception of physical, emotional, social, and cognitive well-being [9]. In two previous prospective pilot studies, survivors of advanced melanoma who were successfully treated with I-O therapy were shown to be at risk of developing emotional distress (based on the Hospital Anxiety and Depression Scale, clinical interview, and psychiatric evaluation), fatigue (based on the Fatigue Severity Scale), subjective cognitive disturbances (based on the Cognitive Failure Questionnaire), and cognitive impairment (based on the Cogstate computerized cognitive test battery), with significant impact on physical, role, emotional, cognitive, and social functioning; fatigue; and global health/quality of life (QLQ), according to the European Organisation for Research and Treatment of Cancer Quality of Life Questionnaire C30 (EORTC QLQ-C30) [10,11]. In another study, long-term survivors of advanced melanoma treated with ipilimumab were found to experience significant impact on fatigue and physical, social, and cognitive functioning compared with matched controls [12]. Expanding on those findings, the current study focused on key issues of HRQoL to detect potential care needs in the adjuvant melanoma population using the following cancer-specific, disease-specific, and generic patient-reported outcomes, respectively: the EORTC QLQ-C30 [13], Functional Assessment of Cancer Therapy—Melanoma (FACT-M) questionnaire [14], and EQ-5D-3L questionnaire [15,16,17]. HRQoL was evaluated at the group level by applying prespecified thresholds of minimally important differences (MIDs) to mean changes in scores from baseline for the EORTC QLQ-C30 [18,19], FACT-M questionnaire [20,21,22], and EQ-5D-3L questionnaire [23]. In addition, HRQoL was evaluated at the individual patient level by applying prespecified thresholds of clinical importance (TCIs) at specific time points for certain EORTC QLQ-C30 subscales [24] that alert clinicians that a health problem is clinically relevant for the clinical encounter. Furthermore, baseline HRQoL scores were compared between the PRESERV MEL study group and the general population to confirm that HRQoL was comparable between the group of disease-free patients with resected melanoma and healthy individuals.

## 2. Materials and Methods

### 2.1. Study Design

Patients in this non-comparative observational study were enrolled prospectively and retrospectively during a 2-year period (January 2019–January 2021) and will be followed for up to 5 years, or until death, loss to follow-up, or withdrawal of consent, whichever occurs first (Supplemental Figure S1). The results reported here are for the second interim analysis (cutoff date 31 December 2021), and none of the patients had reached the planned 5-year follow-up at the time of this interim analysis. Patient selection was based on the systematic sampling technique (i.e., all consecutive eligible patients were included in the study). Inclusion criteria were being 18 years of age or older, having a primary diagnosis of melanoma with lymph node involvement or metastatic disease, having undergone complete resection with no evidence of disease, and having made a decision to treat with adjuvant nivolumab therapy. Exclusion criteria were having a diagnosis of persisting advanced melanoma prior to first administration of nivolumab and being enrolled in an interventional clinical trial involving melanoma treatment. The index date was the date of first administration of adjuvant nivolumab. Patients received adjuvant nivolumab with the intention to treat for up to 12 months per label [5]. Those who received adjuvant nivolumab for more than 12 months were not excluded.

The primary objective of the study was to evaluate RFS over 5 years. Secondary objectives were to evaluate distant metastasis-free survival (DMFS) over 5 years, overall survival over 5 years, baseline demographic and disease characteristics, pattern of use of adjuvant nivolumab therapy, health-care resource utilization (HCRU), HRQoL, and the safety profile for adjuvant nivolumab therapy.

Institutional review board/independent ethics committee approvals/favorable opinions were obtained prior to initiation of the study. In accordance with local regulations, patients provided either written or oral consent to participate in the study prior to enrollment. Informed consent forms were provided in the patient’s local language (i.e., Belgian Dutch, French, German, or English). For retrospectively enrolled patients who were deceased at the beginning of the study, a consent waiver was sought.

### 2.2. Assessments

Median follow-up was defined as the time from index date to study completion date for patients who discontinued early or last visit date for patients who continued. Minimum follow-up was defined as the difference between index date and database lock. RFS was defined as the time from index date to date of disease recurrence, death, or loss to follow-up, whichever occurred first. Time to treatment discontinuation (TTD) was evaluated and defined as the time from index date to date of treatment discontinuation, death, or loss to follow-up, whichever occurred first. Any-cause AEs, treatment-related AEs (TRAEs), and immune-related TRAEs (i.e., those having an immunologic etiology) were reported. AE severity was graded according to the National Cancer Institute Common Terminology Criteria for Adverse Events grading system, version 4.8, as follows: grade 1, mild; grade 2, moderate; grade 3, severe or medically significant but not immediately life-threatening; grade 4, life-threatening; and grade 5, death related to AE. Serious TRAEs were defined as those that resulted in death, were life-threatening, required hospitalization or prolonged an existing hospitalization, resulted in persistent or significant disability/incapacity, or were an important medical event. Time to onset and time to resolution of immune-related TRAEs were evaluated for the following categories: skin, gastrointestinal, hepatobiliary, respiratory, endocrine, and renal. Data for overall survival, DMFS, and HCRU are not presented because follow-up was not long enough for maturity of those data sets.

Among prospectively enrolled patients, HRQoL was evaluated and captured using the EORTC QLQ-C30 [13], FACT-M questionnaire [14], and EQ-5D-3L questionnaire [15,16,17]. Validated, translated (i.e., Belgian Dutch, French, German, or English) versions of the three questionnaires were provided to patients. The EORTC QLQ-C30 is a self-rated, 30-item questionnaire consisting of a global health status/QoL subscale, five functioning subscales (physical, role, emotional, cognitive, and social), three symptom subscales (fatigue, nausea/vomiting, and pain), and six single-item subscales (dyspnea, insomnia, appetite loss, constipation, diarrhea, and financial difficulties) [13]. EORTC QLQ-C30 scores are linearly transformed to range from 0 to 100, with higher scores indicating better HRQoL on the global health status/QoL subscale, better functioning on the functioning subscales, and worse symptoms/problems on the symptom and single-item subscales. The FACT-M questionnaire consists of a 27-item questionnaire that measures physical, social, emotional, and functional well-being (FACT-General [FACT-G]) in addition to a melanoma-specific subscale and a melanoma surgery subscale [14]. The FACT-M questionnaire is relevant to patients in the adjuvant (postsurgical) setting due to the inclusion of the surgery subscale. The FACT-M Trial Outcome Index was assessed and is the sum of physical well-being, functional well-being, and melanoma-specific subscale scores [22]. Higher FACT-M scores indicate better HRQoL. The EQ-5D-3L questionnaire is a general HRQoL instrument consisting of two separate sections that evaluate health utility and overall health status [15,16,17]. The descriptive system in the EQ-5D-3L assesses five dimensions of health (mobility, self-care, usual activities, pain/discomfort, and anxiety/depression), with patient responses converted into a health utility index value based on 1 as full health, 0 as dead, and negative values as a state considered worse than death. In the visual analogue scale (VAS) section of the EQ-5D-3L, respondents rate their own current health on a scale ranging from 0 to 100 (worst to best imaginable health). The EQ-5D-3L health utility index measures population preference-based health status, and the EQ-5D-3L VAS measures a patient’s overall health status.

### 2.3. Statistical Analysis

Median RFS and TTD with associated 95% CIs were estimated using the Kaplan–Meier product limit method. Time to onset and resolution of immune-related TRAEs were evaluated using medians and ranges. Comparisons were made between baseline EORTC QLQ-C30 and EQ-5D-3L mean scores in PRESERV MEL and estimated mean scores from the general population [25,26,27] to determine if baseline scores among disease-free patients with resected melanoma in PRESERV MEL were an adequate point of reference for evaluating the impact of treatment on HRQoL. The PRO questionnaire completion rate was calculated using the number of patients with non-missing PRO data at baseline and at a postbaseline visit, divided by the number of patients eligible for the respective visit.

At the group level, HRQoL was assessed using the EORTC QLQ-C30, FACT-M, and EQ-5D-3L instruments at baseline (prior to the first administration of adjuvant nivolumab) and at 3, 6, 9, 12, 18, and 24 months. Changes from baseline in HRQoL scores were evaluated using a mixed model for repeated measures, with least squares mean changes and associated 95% CIs reported. Clinically meaningful changes from baseline were determined using prespecified thresholds for MIDs for EORTC QLQ-C30 (melanoma-specific and general MIDs) [18,19], FACT-M [20,21,22], and EQ-5D-3L [23] (Supplementary Table S1).

At the individual patient level, HRQoL was assessed using the EORTC QLQ-C30 subscales of emotional, cognitive, and social functioning and fatigue at baseline (prior to first administration of treatment), at 9 months (when most patients were still receiving adjuvant nivolumab treatment), and at 18 months (6 months after discontinuation of the 12-month treatment course). Percentages of patients with scores exceeding TCIs for the subscales of emotional, cognitive, and social functioning (defined as emotional, cognitive, and social impairment, respectively) and fatigue [24] were reported. In a non-protocol, exploratory analysis, individual global health/QLQ scores were evaluated after recovery from grade 3 or 4 TRAEs.

## 3. Results

### 3.1. Patient Disposition and Baseline Characteristics

**Figure 1:**
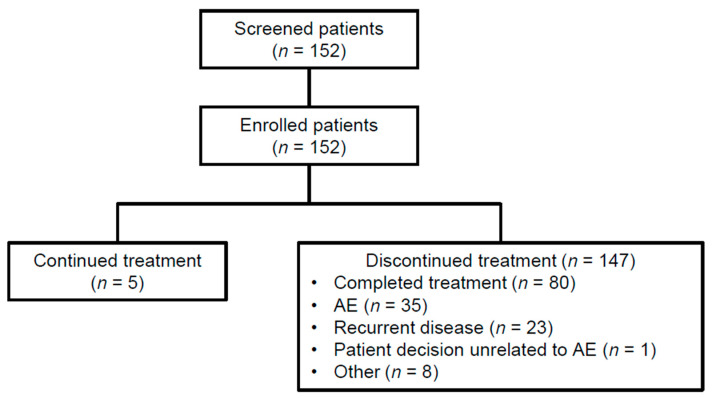
Patient disposition. AE, adverse event.

The study enrolled 152 patients (125 prospective and 27 retrospective) at 15 hospitals in Belgium (14 sites) and Luxembourg (1 site) during a 2-year period (January 2019–January 2021). Patient disposition is shown in Figure 1.

Median age at index date was 60 years (range 29–85), and 53% of patients (*n* = 80) were male (Table 1). The most common primary melanoma subtype was superficial spreading melanoma (45%; *n* = 69), followed by nodular melanoma (24%; *n* = 37). Thirty-eight percent of patients (*n* = 58) had *BRAF* mutant disease, 43% (*n* = 65) had *BRAF* wild-type disease, and 18% (*n* = 27) had missing/unknown *BRAF* status. Eighty-three percent of patients (*n* = 126) had stage III disease, 11% (*n* = 17) had stage IV disease, and 6% (*n* = 9) had other/missing disease stage. Sixty-four percent of patients (*n* = 97) had an Eastern Cooperative Oncology Group performance status of 0 (i.e., fully active). Median time from surgical resection to index date was 1.2 months (range 0.1–14.2). Baseline sociodemographic characteristics are presented in Supplementary Table S2.

### 3.2. Treatment Exposure

All patients received one or more doses of nivolumab. At the time of analysis, 3% of patients (*n* = 5) were still receiving nivolumab and 97% (*n* = 147) had discontinued treatment because of treatment completion (53%, *n* = 80), AEs (23%, *n* = 35), disease recurrence (15%, *n* = 23), patient decision unrelated to AEs (1%, *n* = 1), or other reasons (5%, *n* = 8). Median TTD in the overall study population, patients who discontinued treatment because of AEs, and patients who had disease recurrence were 11.1 months (95% CI: 10.8–11.3), 6.4 months (95% CI: 3.4–8.3), and 5.5 months (95% CI: 2.9–6.9), respectively.

### 3.3. Effectiveness

**Figure 2:**
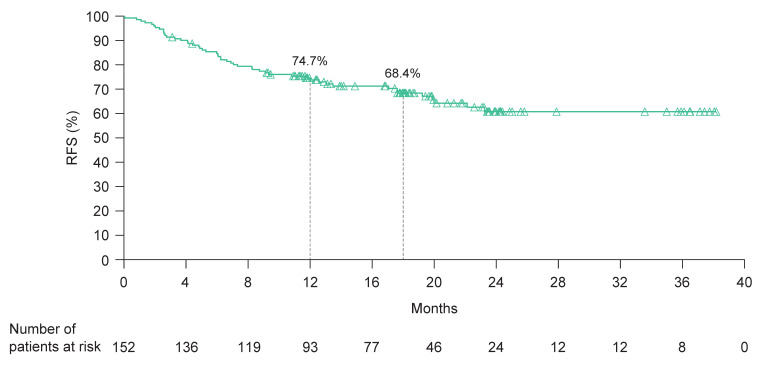
RFS in patients with completely resected stage III or stage IV melanoma treated with adjuvant nivolumab. Patients were indexed to the date of first adjuvant nivolumab dose. RFS, relapse-free survival.

Minimum follow-up was 11.4 months. Median follow-up was 18.5 months (range 3–38). At 12 and 18 months, the RFS rates were 74.7% (95% CI: 66.9–80.9) and 68.4% (95% CI: 60.0–75.5), respectively; median RFS was not reached (Figure 2). During the observed study period, 33% of patients (*n* = 50) experienced disease recurrence, with local recurrence occurring in 10% (*n* = 15) of patients, regional recurrence in 7% (*n* = 11), distant recurrence in 15% (*n* = 23, among which two patients had relapse involving the CNS), and missing recurrence information in 1% (*n* = 1).

### 3.4. Safety

During the observed study period, 96% of patients (*n* = 146) experienced at least one AE, 86% (*n* = 131) experienced at least one TRAE, and 14% (*n* = 21) experienced at least one grade 3 or 4 TRAE (Table 2).

An AE leading to treatment discontinuation was reported in 23% of patients (*n* = 35). The most common any-grade TRAEs were fatigue (49%; *n* = 75), pruritus (24%; *n* = 36), and diarrhea (15%; *n* = 23) (Table 3). Death occurred in 3% of patients (*n* = 5), and none of these deaths was considered related to treatment.

Any-grade immune-related TRAEs were reported in 57% of patients (*n* = 86), and grade 3 or 4 immune-related TRAEs were reported in 11% of patients (*n* = 17). The most common any-grade immune-related TRAEs were fatigue (17%; *n* = 26), pruritus (12%; *n* = 18), and diarrhea (9%; *n* = 14). The most common any-grade immune-related TRAEs of special interest were hepatitis (5%; *n* = 7), pneumonitis (4%; *n* = 6), and adrenal insufficiency (3%; *n* = 4) (Table 4).

**Figure 3:**
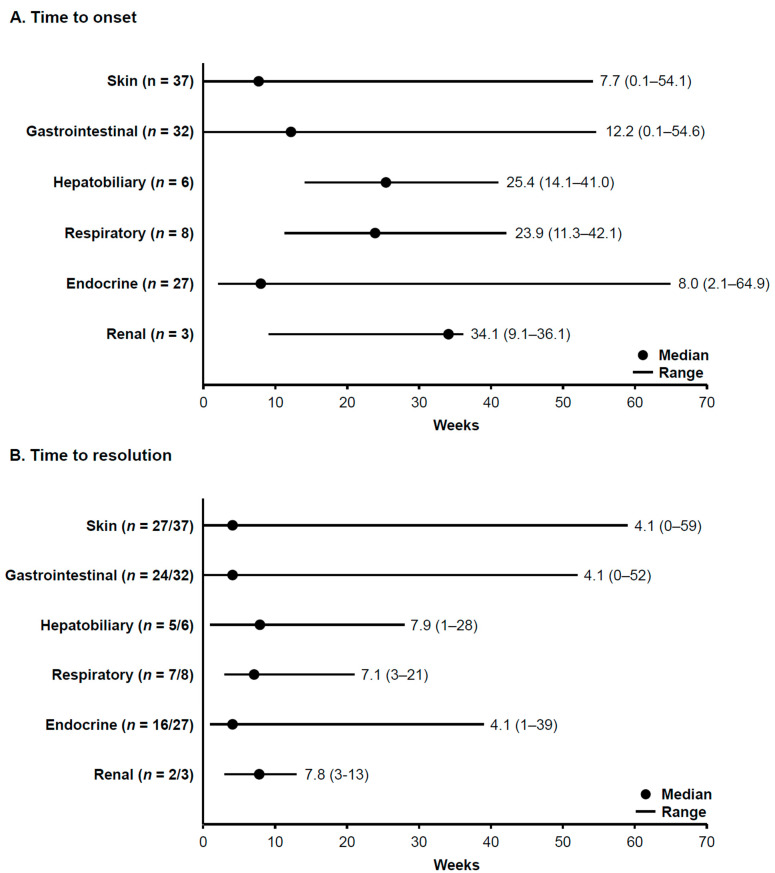
Median (range) time to onset (**A**) and resolution (**B**) of immune-related TRAEs (any grade). TRAE, treatment-related adverse event.

Median times to onset of immune-related TRAEs (any grade) were 7.7 weeks for skin TRAEs (*n* = 37), 12.2 weeks for gastrointestinal TRAEs (*n* = 32), 25.4 weeks for hepatobiliary TRAEs (*n* = 6), 23.9 weeks for respiratory TRAEs (*n* = 8), 8.0 weeks for endocrine TRAEs (*n* = 27), and 34.1 weeks for renal TRAEs (*n* = 3) (Figure 3A). Resolution rates for immune-related TRAEs (any grade) were 73% (27/37) for skin TRAEs, 75% (24/32) for gastrointestinal TRAEs, 83% (5/6) for hepatobiliary TRAEs, 88% (7/8) for respiratory TRAEs, 59% (16/27) for endocrine TRAEs, and 67% (2/3) for renal TRAEs. Median time to resolution of immune-related TRAEs (any grade) was 4.1 weeks for skin TRAEs, 4.1 weeks for gastrointestinal TRAEs, 7.9 weeks for hepatobiliary TRAEs, 7.1 weeks for respiratory TRAEs, 4.1 weeks for endocrine TRAEs, and 7.8 weeks for renal TRAEs (Figure 3B).

A total of 27 patients experienced a serious TRAE (*n* = 11) and/or a grade 3 or 4 TRAE (*n* = 21). Five of the 27 patients experienced a serious TRAE that was also a grade 3 or 4 TRAE. In 19 of the 27 patients, HRQoL data were available at the predefined time points before the occurrence of and after the recovery from a serious TRAE (7/11) or grade 3 or 4 TRAE (12/21).

### 3.5. Health-Related Quality of Life

Among the 125 prospectively enrolled patients, patient-reported outcome questionnaire completion rates were 90–94% at baseline, 76–80% at 12 months, and 78–81% at 24 months (Supplementary Table S3). No clinically relevant differences were found in the mean scores for the EORTC QLQ-C30 at baseline between the study group and the general population (a 10-point difference indicated a clinically relevant difference [19,25]) for any subscale, suggesting that baseline scores among disease-free patients with resected melanoma in PRESERV MEL were an adequate point of reference for evaluating the impact of treatment on HRQoL (Table 5). Mean baseline scores for the EQ-5D-3L VAS and utility index were numerically similar between enrolled patients and the general population (Table 5) [26,27].

**Figure 4:**
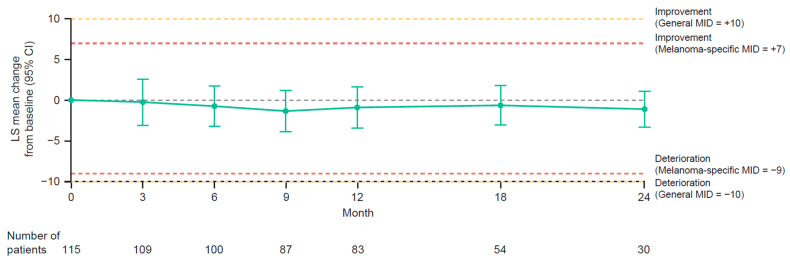
LS mean changes from baseline in EORTC QLQ-C30 global health/QoL scores in patients with completely resected stage III or stage IV melanoma treated with adjuvant nivolumab. CI, confidence interval; EORTC QLQ-C30, European Organisation for Research and Treatment of Cancer Quality of Life Questionnaire C30; LS, least squares; MID, minimally important difference; QoL, quality of life.

In the group-level analysis, least squares mean changes from baseline in EORTC QLQ-C30 global health/QoL scores were stable (i.e., close to baseline values) and did not exceed thresholds for MIDs (melanoma-specific or general) at any time point (Figure 4) [18,19]. Least squares mean changes from baseline for the five functioning subscales (physical, role, emotional, cognitive, and social), three symptom subscales (fatigue, nausea/vomiting, and pain), and six single-item subscales (dyspnea, insomnia, appetite loss, constipation, diarrhea, and financial difficulties) of the EORTC QLQ-C30 were stable (i.e., close to baseline values) and did not exceed threshold for MIDs (melanoma-specific or general) at any time point [18,19]. Although cognitive functioning mean scores remained stable, they decreased (deteriorated) and approached the prespecified thresholds for MIDs.

**Figure 5:**
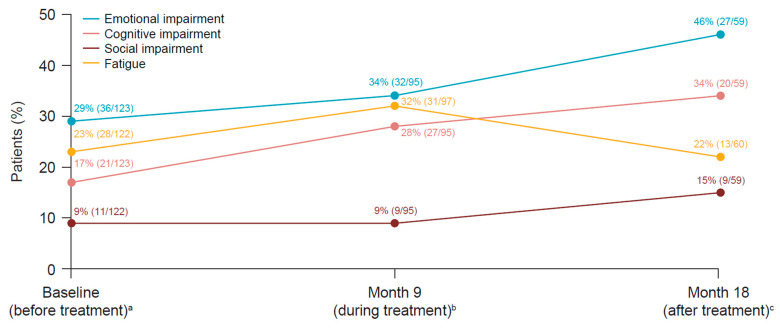
Patients reporting emotional, cognitive, and social impairment and fatigue on the EORTC QLQ-C30 as defined by scores exceeding the TCIs for individual patients according to [24]. ^a^ Prior to the first administration of adjuvant nivolumab treatment. ^b^ Time point at which most patients were still receiving adjuvant nivolumab treatment. ^c^ 6 months after discontinuation of the 12-month course of adjuvant nivolumab treatment. TCI, threshold of clinical importance.

In the individual patient-level analysis for EORTC QLQ-C30 subscales, the percentage of patients who reported scores that exceeded the TCI [24] for fatigue and emotional and cognitive impairment increased during treatment (at 9 months) compared with baseline (prior to first administration of treatment). After treatment cessation (at 18 months), the percentage of patients who reported scores that exceeded the TCI for fatigue decreased and the percentage of patients who reported scores that exceeded TCIs for emotional, cognitive, and social impairment increased compared with during treatment (at 9 months) (Figure 5).

Most patients with emotional impairment at 9 and 18 months did not experience disease recurrence (29/32 [91%] and 24/27 [89%], respectively). In a non-protocol, exploratory analysis, stabilization or improvement (after an initial decline in some cases) in individual global health/QLQ scores with the EORTC QLQ-C30 was documented after recovery from grade 3 or 4 TRAEs in 12 patients who completed their patient-reported outcome questionnaires during and after those TRAEs.

**Figure 6:**
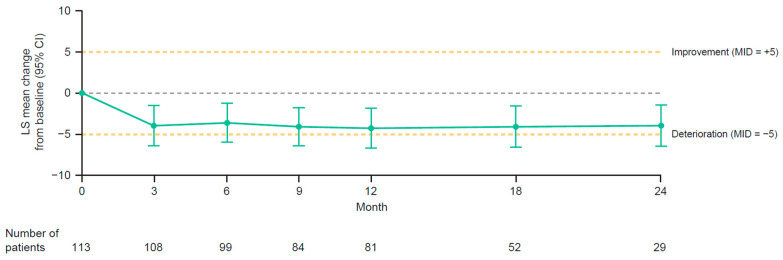
Least squares mean changes from baseline in FACT-M total scores in patients with completely resected stage III or stage IV melanoma treated with adjuvant nivolumab. CI, confidence interval; FACT-M, Functional Assessment of Cancer Therapy—Melanoma; LS, least squares; MID, minimally important difference.

At any time point, least squares mean changes from baseline in FACT-M total scores were stable (i.e., close to baseline values), not exceeding thresholds for MIDs [20] (Figure 6). Although remaining within the thresholds for MIDs, FACT-M total scores deteriorated during the first 3 months of treatment and later stabilized. Least squares mean changes from baseline on the FACT-M melanoma subscale, FACT-M Trial Outcome Index, and FACT-M physical, social/family, emotional, and functional well-being scores were stable, not exceeding thresholds for MIDs [20,21,22] at any time point. From 3 months onward, least squares mean changes from baseline in FACT-M melanoma surgery subscale scores improved, exceeding the thresholds for MID [21].

At any time point, least squares mean changes from baseline in EQ-5D-3L VAS and utility index scores were stable (i.e., close to baseline values) and did not exceed thresholds for MIDs [23].

## 4. Discussion

Results of the PRESERV MEL confirm the real-world effectiveness and safety of adjuvant nivolumab in patients with completely resected stage III or stage IV melanoma. PRESERV MEL included a cohort of 125 prospectively enrolled patients in Belgium and Luxembourg, representing one of the largest real-world HRQoL datasets reported for an I-O therapy as adjuvant treatment for patients with completely resected stage III or stage IV melanoma. Within this cohort, cancer-specific, disease-specific, and generic HRQoL were maintained during and after treatment. Results from PRESERV MEL were generally consistent with those from the two phase III RCTs evaluating adjuvant nivolumab in patients with completely resected melanoma: CheckMate 238 and CheckMate 915 [1,4]. Of note, completion rates of captured HRQoL data were higher in this study than in the RCTs.

Baseline characteristics of patients in PRESERV MEL were largely consistent with those of patients in CheckMate 238, which enrolled patients with completely resected stage IIIB, stage IIIC, or stage IV melanoma (per AJCC-7) [1]. Among patients treated with nivolumab in PRESERV MEL and CheckMate 238, respectively, median age was 60.0 and 56.0 years, 53% and 57% were male, and 38% and 41% had *BRAF* mutant disease [1]. However, a higher percentage of patients had stage IIIA disease (as per AJCC-8) in PRESERV MEL than in CheckMate 238 (18% vs. 1%) [28]. This finding may reflect the willingness in recent years to use adjuvant nivolumab in patients with resected stage IIIA melanoma, regardless of the sentinel node tumor burden, as this is an approved indication, notwithstanding the use of more restrictive eligibility criteria in prospective trials. Furthermore, enrollment for CheckMate 238 was conducted in 2015 (compared with 2019–2021 for PRESERV MEL), and since that time, real-world evidence has emerged suggesting that adjuvant nivolumab treatment may provide benefit to patients with resected stage IIIA melanoma [29,30,31,32].

Effectiveness with adjuvant nivolumab in PRESERV MEL was comparable to efficacy in CheckMate 238 and CheckMate 915 [1,4,33], further supporting the use of adjuvant nivolumab for patients with resected advanced melanoma. The 24-month RFS rates were 60.8% in PRESERV MEL (minimum follow-up 11.4 months), 62.6% in CheckMate 238 (minimum follow-up 24 months) [33], and 63.2% in CheckMate 915 (minimum follow-up 23.7 months) [4]. Despite the enrollment of patients with stage IIIA disease, who have a relatively good prognosis, in PRESERV MEL (18% of the study group), but not in CheckMate 238 or CheckMate 915, the 24-month RFS rate was slightly lower in PRESERV MEL than in CheckMate 238 or CheckMate 915. The difference in RFS rates between PRESERV MEL and the two RCTs may have been due to differences in baseline characteristics.

Safety was also similar in PRESERV MEL and the CheckMate 238 and CheckMate 915 trials [1,4], suggesting that adjuvant nivolumab is equally tolerated in clinical practice and investigational studies. Grade 3 or 4 TRAEs were reported in 14% of patients treated with nivolumab in PRESERV MEL and CheckMate 238, and in 13% of patients treated with nivolumab in CheckMate 915 [4]. The most common TRAEs in PRESERV MEL, CheckMate 238, and CheckMate 915 were fatigue (49%, 34%, and 30%, respectively), pruritus (24%, 23%, and 21%, respectively), and diarrhea (15%, 24%, and 20%, respectively). Interestingly, the incidence of any-grade fatigue was similar between adjuvant nivolumab and placebo (20% vs. 20%) and adjuvant pembrolizumab (an anti–PD-1 I-O therapy) and placebo (20% vs. 18%) in separate phase III trials (CheckMate 76K and KEYNOTE-716, respectively) involving patients with resected stage IIB or stage IIC melanoma [34,35]. Despite similar safety profiles for adjuvant nivolumab across the studies, the rate of treatment discontinuation due to TRAEs was higher in PRESERV MEL (19%) than in CheckMate 238 or CheckMate 915 (8% and 10%) [1,4]. The higher discontinuation rate in PRESERV MEL may have been related to less stringent treatment requirements, less patient motivation to remain in treatment, and/or more cautious decision-making for stopping treatment in real-world clinical practice than in RCTs. However, less exposure to study treatment in PRESERV MEL than in CheckMate 238 or CheckMate 915 did not translate into less clinical benefit, as effectiveness in PRESERV MEL was similar to efficacy in the two RCTs.

PRESERV MEL is the first real-world study to evaluate HRQoL in patients with resected melanoma who were treated with adjuvant nivolumab. Baseline patient-reported outcome scores for patients enrolled in PRESERV MEL were similar to scores for the general population, suggesting that baseline scores in PRESERV MEL were an adequate point of reference for evaluating the impact of treatment on HRQoL. The patient-reported outcomes analysis was also supported by very high questionnaire completion rates at baseline (90–94%) and at 24 months (78–81%). Cancer-specific, disease-specific, and generic HRQoL outcomes remained close to baseline levels after initiation of adjuvant nivolumab, with no clinically meaningful change over time for symptoms, functioning, or global health/QLQ, except for a clinically meaningful improvement in FACT-M surgery subscale scores. Given that the FACT-M surgery subscale addresses the impact of immediate postoperative swelling from surgery, those scores were expected to improve over time. Results for HRQoL in PRESERV MEL are consistent with those in CheckMate 238 and CheckMate 915, in which there were no clinically meaningful differences (based on prespecified thresholds for MIDs) from baseline in HRQoL in patients treated with up to 1 year of adjuvant nivolumab treatment [1,4]. Findings of PRESERV MEL are also in line with those of a systematic review and meta-analysis involving 34 RCTs and 18,709 patients with various cancers, suggesting that I-O treatments do not significantly affect patient-reported HRQoL [36]. Furthermore, results from clinical trials in advanced melanoma have shown that OS improvement with anti–PD-1 therapy is associated with HRQoL benefit when compared with chemotherapy [37]. Additionally, in a cross-sectional survey and chart review analysis, patients (*n* = 90) with advanced melanoma who survived > 1 year after initiating I-O therapy frequently reported fatigue, but otherwise demonstrated moderate symptom burden and good HRQoL [38].

Although remaining within the thresholds for MIDs at all time points, EORTC QLQ-C30 global health/QoL scores in PRESERV MEL deteriorated slightly during the first 9 months of treatment and later improved, approaching baseline level. The early decline and subsequent improvement/stabilization in EORTC QLQ global health/QLQ scores may have been due to the onset and resolution of immune-related TRAEs. In PRESERV MEL, stabilization or improvement (after an initial decline in some cases) in individual EORTC QLQ-C30 global health/QLQ scores was documented after recovery from serious and/or grade 3 or 4 TRAEs in 12 patients who completed their patient-reported outcome questionnaires during and after those TRAEs.

Mean FACT-M total scores deteriorated during the first 3 months of treatment and later stabilized while remaining within the thresholds for MIDs at all time points. As the FACT-M total score is the sum of the FACT-G and the FACT-M, this decline may be explained by an improvement in symptoms related to surgery.

Even though fatigue was the most common any-grade TRAE in PRESERV MEL, EORTC QLQ-C30 fatigue mean scores remained stable over time and did not exceed prespecified thresholds for MIDs. Moreover, when considering the TCI [24] in the individual patient-level analysis, fatigue was reported by 23% of the patients at baseline as a clinically important symptom, increasing to 32% of the patients during treatment (9 months) and decreasing to 22% of the patients after stopping treatment (18 months), suggesting that fatigue associated with adjuvant nivolumab treatment did not have a negative impact on HRQoL as measured by the EORTC QLQ-C30 instrument. Of interest, clinicians reported fatigue more frequently as a TRAE (49%) than patients did as a symptom of clinical importance based on TCI (32%) [24]. Fatigue is one of the most frequent complaints among patients with cancer, and it has a multifactorial etiology that includes being a symptom of the disease itself, an AE of both cancer therapies and other medications, and a psychological complication of cancer and its treatment [39]. I-O therapy is typically associated with fatigue, although less so than with chemotherapy [39]. Specific factors causing fatigue in patients receiving I-O therapy are generally unknown, but in certain instances, other TRAEs, such as cardiac, pulmonary, and endocrine TRAEs, may precipitate fatigue [40]. It should be noted that although fatigue was classified as a TRAE in this study, it may be more appropriately considered a treatment-emergent AE, given that its relationship to I-O therapy is unclear.

Impaired objective and subjective cognitive functioning are also common in patients with cancer and may be related to the disease itself, cancer treatments, fatigue or emotional distress, and interference with the ability to return to work [41]. In PRESERV MEL, mean scores for EORTC QLQ-C30 cognitive functioning (reflecting subjective cognitive complaints) remained stable, but decreased (deteriorated) and approached the prespecified thresholds for MIDs. When examining cognitive functioning on an individual patient level, there was an increase in the percentage of patients reporting difficulties with cognitive functioning exceeding the TCI [24] during treatment (9 months) and after stopping treatment (18 months). Subjective cognitive complaints are multifactorial and can be provoked by the cancer itself, cancer treatment, fatigue, and emotional and sleep disturbances [41]. Information on the impact of I-O therapy on objective cognitive impairment in patients with cancer is still scarce, but this cognitive impairment could potentially be related to neuroendocrine or immune factors [10,11,42]. Therefore, further research is needed that specifically focuses on objective and subjective cognitive function during and after administration of I-O therapy. An increased percentage of patients reported emotional and social impairment exceeding TCIs [24] at 18 months, when all patients were off treatment. This finding was not related to recurrence of disease. Impaired cognitive, social, and emotional functioning after stopping treatment is in line with other studies in the metastatic setting [10,11,43]. These results are of potential clinical interest in terms of defining the need for supportive care, especially considering that the PRESERV MEL study was conducted in a real-world setting. Therefore, future studies should include continued monitoring of cognitive, emotional, and social function after adjuvant treatment is stopped and at disease recurrence. These observations justify future interventional studies to diagnose and treat such dysfunctions after the end of adjuvant therapy.

This study has certain limitations. Firstly, this was a non-comparative, observational, descriptive study that did not control for multiple comparisons. In addition, results of this study may have been influenced by differences in characteristics between the baseline population and patients remaining on treatment at later time points, who were more likely to have responded to or tolerated treatment. Although patient-reported outcome completion rates (calculated based on the number of patients at each time point) were high throughout PRESERV MEL, the number of patients completing the patient-reported outcome questionnaires decreased at later time points. This decrease may have been due in part to patients having been enrolled consecutively; thus, some patients had not yet reached the later time points at the time of the analysis. In patient-reported outcome studies in general, the effect of treatment on HRQoL may be underestimated because of low patient numbers at later time points resulting from discontinuation related to disease progression, AEs, and death [44]. Additionally, currently available patient-reported outcomes were designed before the introduction of I-O therapy and thus may not fully delineate the effect of I-O therapy on HRQoL. However, the HRQoL analysis was strengthened by high patient-reported outcome completion rates among eligible patients throughout the study period and by including patient-reported outcome data during and after serious and grade 3 or 4 TRAEs.

## 5. Conclusions

In summary, interim results of the PRESERV MEL study, which was conducted in 152 patients in Belgium and Luxembourg, confirm the real-world effectiveness and safety of adjuvant nivolumab in patients with completely resected stage III or stage IV melanoma and suggest that HRQoL with adjuvant nivolumab treatment is maintained. Patients’ symptoms, functioning, and overall HRQoL remained stable over the course of treatment, as measured by cancer-specific, disease-specific, and generic patient-reported outcomes. Six months after the maximum treatment duration (at 18 months), some patients reported clinical important emotional, cognitive, and social impairment, which needs to be further investigated and justifies offering tailored supportive care/treatment for these patients. Findings from this study were generally consistent with those from the phase III CheckMate 238 and CheckMate 915 trials [1,4], further supporting the use of adjuvant nivolumab as a standard of care for patients with completely resected stage III or stage IV melanoma.

## Figures and Tables

**Table 1:** Baseline demographic and disease characteristics.

Characteristic	Adjuvant Nivolumab (*n* = 152)
Median age, years (range)	60.0 (29–85)
Male, *n* (%)	80 (53)
Primary melanoma subtype, *n* (%) ^a^	
Superficial spreading	69 (45)
Nodular	37 (24)
Acral lentiginous	6 (4)
Lentigo maligna	2 (1)
Not otherwise specified	29 (19)
Missing	9 (6)
Primary melanoma location, *n* (%)	
Limbs	86 (57)
Trunk	47 (31)
Head/neck	10 (7)
Missing	9 (6)
Primary melanoma resection, *n* (%)	143 (94)
SLNB performed, *n* (%)	127 (84)
≥1 positive sentinel lymph node, *n* (%)	
Yes	93 (61)
No	31 (20)
Missing	28 (18)
Complete lymph node dissection, *n* (%)	
Yes	53 (35)
No	97 (64)
Missing	2 (1)
Complete lymph node dissection in patients with non-occult disease, *n* (%)	
Yes	21 (14)
No	18 (12)
Clinically occult only lymph node involvement, n (%)	74 (49)
Resected stage at baseline (per AJCC-8), *n* (%)	
IIIA	28 (18)
IIIB	41 (27)
IIIC	51 (34)
IIID	6 (4)
IV	17 (11)
Other	8 (5)
Missing	1 (1)
Tumor ulceration patients with stage III disease, *n* (%)	
Yes	48 (32)
No	69 (45)
Missing	9 (6)
SLN metastasis with longest diameter < 1 mm, *n* (%)	
Yes	13 (9)
No	63 (41)
Missing	51 (34)
Stage IIIA with a longest diameter of the sentinel lymph node metastasis < 1 mm, *n* (%)	
Yes	10 (7) ^b^
No	12 (8)
Missing	6 (4)
*BRAF* status, *n* (%)	
Mutant (positive)	58 (38)
Wild-type (negative)	65(43)
Unknown	2 (1)
Missing	27 (18)
ECOG performance status, *n* (%)	
0	97 (64)
1	14 (9)
2	1 (1)
Missing	40 (26)
Previous enrollment in an interventional study, *n* (%)	2 (1)
Median time from surgical resection to index date, months (range)	1.2 (0.1–14.2)

^a^ No cases of desmoplastic or mucosal melanoma. ^b^ 36% of patients with stage IIIA disease (10/28). AJCC-8, American Joint Committee on Cancer, *Cancer Staging Manual*, eighth edition; ECOG, Eastern Cooperative Oncology Group; SLN, sentinel lymph node; SLNB, SLN biopsy.

**Table 2:** Safety summary.

	Adjuvant Nivolumab (*n* = 152)	
	Any Grade, *n* (%)	Grade 3 or 4, *n* (%)
Any AE	146 (96)	49 (32)
TRAE	131 (86)	21 (14)
Any AE leading to treatment discontinuation	35 (23)	15 (10)
TRAE leading to treatment discontinuation	29 (19)	12 (8)

AE, adverse event; TRAE, treatment-related adverse event.

**Table 3:** Most common (≥4% of patients) TRAEs ^a^.

	Adjuvant Nivolumab (*n* = 152)	
	Any Grade, *n* (%)	Grade 3 or 4, *n* (%) ^b^
TRAE	131 (86)	21 (14)
Fatigue	75 (49)	1 (1)
Pruritus	36 (24)	0
Diarrhea	23 (15)	1 (1)
Hypothyroidism	22 (14)	0
Nausea	16 (11)	0
Hyperthyroidism	14 (9)	0
Dry mouth	12 (8)	0
Rash	12 (8)	0
Dry skin	9 (6)	0
Arthralgia	9 (6)	0
Colitis	8 (5)	2 (1)
Myalgia	8 (5)	1 (1)
Headache	8 (5)	0
Hepatitis	7 (5)	2 (1)

^a^ Reported between the first nivolumab dose and ≤2 years after the last dose. ^b^ Grade 3 or 4 TRAEs not listed included pneumonitis (*n* = 3) and psoriasis, skin reaction, stomatitis, pancreatitis, adrenal insufficiency, osteoarthritis, myositis, elevated alanine aminotransaminase level, elevated aspartate aminotransaminase level, decreased cortisol, abnormal liver function test, aseptic meningitis, infusion-related reaction, and myocarditis (*n* = 1 each). TRAE, treatment-related adverse event.

**Table 4:** Immune-related TRAEs of special interest.

	Total (*n* = 152)	
	Any Grade, *n* (%)	Grade 3 or 4, *n* (%)
Hepatitis	7 (5)	2 (1) ^a^
Pneumonitis	6 (4)	3 (2) ^b^
Adrenal insufficiency	4 (3)	1 (1) ^c^
Renal disorders	3 (2)	0
Meningitis aseptic	1 (1)	1 (1) ^d^
Myasthenia gravis	1 (1)	0
Myocarditis	1 (1)	1 (1) ^d^

^a^ One case resolved; one case resolving. ^b^ Two cases resolved; one case resolved with sequelae. ^c^ Resolved with sequelae. ^d^ Resolved. TRAE, treatment-related adverse event.

**Table 5:** Comparison of baseline EORTC QLQ-C30 and EQ-5D-3L scores in PRESERV MEL with estimated scores for the general population.

Subscale	Mean Score (SD)		Difference
	PRESERV MEL (*n* = 117) ^a^	General Population	
EORTC QLQ-C30			
Global health status/QoL	73.8 (19.8)	66.1 (21.7) ^b^	7.7 (4.1 to 11.4)
Physical functioning	88.5 (17.4)	85.1 (18.9) ^b^	3.4 (0.2 to 6.6)
Role functioning	80.3 (27.8)	84.3 (24.6) ^b^	−4.0 (−9.0 to 1.1)
Emotional functioning	78.3 (22.5)	74.2 (24.7) ^b^	4.1 (0.1 to 8.2)
Cognitive functioning	89.6 (19.7)	84.8 (21.3) ^b^	4.8 (1.2 to 8.4)
Social functioning	87.9 (20.8)	86.2 (24.1) ^b^	1.7 (−2.1 to 5.5)
Fatigue	24.9 (24.4)	29.5 (25.5) ^b^	−4.6 (−9.1 to −0.2)
Nausea/vomiting	1.7 (7.7)	5.9 (16.0) ^b^	−4.2 (−5.6 to −2.8)
Pain	19.8 (24.9)	23.5 (27.1) ^b^	−3.7 (−8.2 to 0.8)
Dyspnea	7.4 (18.6)	15.9 (24.6) ^b^	−8.5 (−11.9 to −5.1)
Insomnia	26.5 (30.8)	26.6 (30.3) ^b^	−0.1 (−5.7 to 5.5)
Appetite loss	10.3 (21.6)	10.0 (21.6) ^b^	0.3 (−3.7 to 4.1)
Constipation	10.1 (21.2)	12.5 (23.3) ^b^	−2.4 (−6.3 to 1.4)
Diarrhea	5.7 (16.0)	9.5 (20.9) ^b^	−3.8 (−6.7 to −0.9)
Financial difficulties	4.8 (17.1)	10.6 (23.6) ^b^	−5.8 (−8.9 to −2.6)
EQ-5D-3L			
VAS	79.5 (17.3)	Female: 76.7 (0.8) ^c^; Male: 78.6 (0.8) ^c^	NA
Utility index	0.82 (0.18)	0.79 (95% CI: 0.786–0.799) ^d^	NA

^a^ Baseline scores; *n* = 115 for global health status/QoL; *n* = 117 for the scales. ^b^ Derived from [25]. ^c^ Derived from [26]. ^d^ Derived from [27]. EORTC QLQ-C30, European Organisation for Research and Treatment of Cancer Quality of Life Questionnaire C30; NA, not available; QoL, quality of life; SD, standard deviation; VAS, visual analogue scale.

## Data Availability

BMS policy on data sharing may be found at https://www.bms.com/researchers-and-partners/clinical-trials-and-research/disclosure-commitment.html (accessed on 26 September 2022).

## Supplementary Materials

The following supporting information can be downloaded at: https://www.mdpi.com/article/10.3390/cancers15194823/s1, Table S1. MIDs for HRQoL Assessments. Table S2. Baseline Sociodemographic Questionnaire Results. Table S3. PRO Completion Rates. Figure S1. PRESERV MEL study design.
