# Supplementary material for: Real-World Effectiveness, Safety, and Health-Related Quality of Life in Patients Receiving Adjuvant Nivolumab for Melanoma in Belgium and Luxembourg: Results of PRESERV MEL
Source: Cancers (Basel). 2023 Sep 30;15(19):4823. doi: 10.3390/cancers15194823 (PMC10572061; doi:10.3390/cancers15194823)

Supplement

Real-World Effectiveness, Safety, and Health-Related Quality of Life in Patients Receiving Adjuvant Nivolumab for Melanoma in Belgium and Luxembourg: Results of PRESERV MEL

Anne Rogiers[1,2,3], Laurence Willemot[4], Laura McDonald[5], Hilde Van Campenhout[4], Guy Berchem[6], Celine Jacobs[7], Nathalie Blockx[8], Andrée Rorive[9], Bart Neyns[2,3]

[1]Centre Hospitalier Universitaire Brugmann, Brussels, Belgium
[2]Universitair Ziekenhuis Brussel, Brussels, Belgium
[3]Vrije Universiteit Brussel, Brussels, Belgium
[4]Bristol Myers Squibb, Braine L'Alleud, Belgium
[5]Bristol Myers Squibb, Uxbridge, United Kingdom
[6]Centre Hospitalier de Luxembourg, University of Luxembourg, Luxembourg
[7]Universitair Ziekenhuis Gent, Gent, Belgium
[8]Ziekenhuis Netwerk Antwerpen Middelheim, Antwerp, Belgium
[9]Centre Hospitalier Universitaire de Liège Sart-Tilman, Liège, Belgium

Supplemental Table S1. MIDs for HRQoL Assessments.

Scale/Subscale	MID	
	**Improvement**	**Deterioration**
EORTC QLQ-C30		
Global health status/QoL	+7 (melanoma-specific)[a]<br>+10 (general)[b]	-9 (melanoma-specific)[a]<br>-10 (general)[b]
Physical functioning	+6 (melanoma-specific)[a]	-6 (melanoma-specific)[a]
Role functioning	+13 (melanoma-specific)[a]	-6 (melanoma-specific)[a]
Emotional functioning	+6 (melanoma-specific)[a]	-6 (melanoma-specific)[a]
Cognitive functioning	NA (melanoma-specific)[a]	-6 (melanoma-specific)[a]
Social functioning	+8 (melanoma-specific)[a]	-7 (melanoma- specific)[a]
Fatigue	-8 (melanoma-specific)[a]	+10 (melanoma-specific)[a]
Nausea/vomiting	-7 (melanoma-specific)[a]	+7 (melanoma-specific)[a]
Pain	-8 (melanoma-specific)[a]	+4 (melanoma-specific)[a]
Dyspnea	-7 (melanoma-specific)[a]	NA[c]
Insomnia	-4 (melanoma specific)[a]	NA (melanoma-specific)[a]
Appetite loss	-12 (melanoma-specific)[a]	+18 (melanoma-specific)[a]
Constipation	NA[c]	NA[c]
Diarrhea	NA[c]	+5 (melanoma-specific)[a]
Financial difficulties	NA[c]	NA[c]
FACT-M		
Total score	+5[d]	-5[d]
Melanoma subscale	+3[e]	-3[e]
TOI	+7	-7
Physical well-being	+2.5[f]	-2.5[f]
Social/family well-being	NA[c]	NA[c]
Emotional well-being	+2[f]	-2[f]
Functional well-being	+2.5[f]	-2.5[f]
Melanoma surgery subscale	+1.5[e]	-1.5[e]
EQ-5D-3L		
Health utility index	+0.08[g]	-0.08[g]
EQ-VAS	+7[g]	-7[g]

[a]Musoro JZ, Bottomley A, Coens C, et al. Interpreting European Organisation for Research and Treatment for Cancer Quality of life Questionnaire core 30 scores as minimally importantly different for patients with malignant melanoma. *Eur J Cancer*. 2018;104:169–181.
[b]Osoba D, Rodrigues G, Myles J, Zee B, Pater J. Interpreting the significance of changes in health-related quality-of-life scores. *J Clin Oncol*. 1998;16(1):139–144.
[c]NA indicates that MIDs have not been reported for those scales.
[d]Bharmal M, Nolte S, Henry-Szatkowski M, Hennessy M, Schlichting M. Update on the psychometric properties and minimal important difference (MID) thresholds of the FACT-M questionnaire for use in treatment-naïve and previously treated patients with metastatic Merkel cell carcinoma. *Health Qual Life Outcomes*. 2020;18(1):145.
[e]Askew RL, Xing Y, Palmer JL, Cella D, Moye LA, Cormier JN. Evaluating minimal important differences for the FACT-Melanoma quality of life questionnaire. *Value Health*. 2009;12(8):1144-1150.
[f]Webster K, Cella D, Yost K. The Functional Assessment of Chronic Illness Therapy (FACIT) Measurement System: properties, applications, and interpretation. *Health Qual Life Outcomes*. 2003;1:79.

[g]Pickard AS, Neary MP, Cella D. Estimation of minimally important differences in EQ-5D utility and VAS scores in cancer. *Health Qual Life Outcomes*. 2007;5:70.

EORTC QLQ-C30, European Organisation for Research and Treatment of Cancer Quality of Life Questionnaire C30; VAS, visual analogue scale; FACT-M, Functional Assessment of Cancer Therapy-Melanoma; HCRU, healthcare resource utilization. HRQoL health-related quality of life; MID, minimally important difference; NA, not available; NA, not available; QoL, quality of life; TOI, Trial Outcome Index.

Supplemental Table S2. Baseline Sociodemographic Questionnaire Results.

**Characteristic, *n* (%)**	**Adjuvant nivolumab (*n* = 152)**
What is your highest level of qualification?	
Primary school	11 (9)
Lower secondary school (general/technical)	13 (10)
Higher secondary school (general/technical)	33 (26)
Professional education (vocational)	16 (13)
High school/Bachelor’s degree	24 (19)
University/Master’s degree	16 (13)
Missing	12 (10)
What is your civil status?	
Married or living together	86 (69)
Divorced and single	8 (6)
Divorced and remarried	5 (4)
Single	10 (8)
Widow	9 (7)
Missing	7 (6)
Number of children	
0	21 (17)
1	26 (21)
2	39 (31)
3	25 (20)
4	7 (6)
5	0
> 5	2 (2)
Missing	5 (4)
What is your social status?	
Employee	35 (28)
Laborer	16 (13)
Independent	13 (10)
Retired	44 (35)
Disabled	1 (1)
OCMW/CPAS support	0
Other	11 (9)
Are you on sick leave at the moment?	
No	60 (48)
Yes	33 (26)
Yes part-time	4 (3)
Not applicable	22 (18)
Missing	6 (5)
Do you have any professional issues or complaints?	
No problem	81 (65)
Stress at work	11 (9)
Job insecurity	1 (1)
Other	11 (9)
Missing	21 (17)

**Characteristic, *n* (%)**	**Adjuvant nivolumab (*n* = 152)**
Do you have any financial problems?	
No	102 (82)
Yes	4 (3)
I need to track my spending	14 (11)
Missing	5 (4)
Do you have any familial issues or complaints?	
No	109 (87)
Yes	11 (9)
Relational problems	3 (2)
Worries about my partner related to my illness	3 (2)
Worries about my children related to my illness	1 (1)
Other worries not related to my illness	4 (3)
Missing	5 (4)
Do you take any sleep medication, sedatives, or antidepressants?	
No	98 (78)
Yes	22 (18)
Missing	5 (4)
Have you experienced any of the following problems in the past?	
No problem	85 (68)
Depression	10 (8)
Burn-out	3 (2)
Drinking problem	1 (1)
Anxiety	7 (6)
Panic attacks	1 (1)
Phobia	1 (1)
Psychosis	0
Traumatic events	6 (5)
Several problems	1 (1)
Other	5 (4)
Missing	5 (4)
Mental attitude	
Optimistic	95 (76)
Pessimistic	21 (17)
Missing	9 (7)

CPAS, Centre public d’action sociale; OCMW, Openbaar centrum voor maatschappelijk welzijn.

Supplemental Table S3. PRO Completion Rates.[a]

Time Point	EORTC QLQ-C30,[b] *n*/*N* (%)	FACT-M,[c] *n*/*N* (%)	EQ-5D-3L,[d] *n*/*N* (%)
Baseline	115/125 (92)	113/125 (90)	117/125 (94)
3 months	109/124 (88)	108/124 (87)	111/124 (90)
6 months	100/118 (85)	99/118 (84)	102/118 (86)
9 months	87/111 (78)	84/111 (76)	87/111 (78)
12 months	83/107 (78)	81/107 (76)	86/107 (80)
18 months	54/69 (78)	52/69 (75)	56/69 (81)
24 months	30/37 (81)	29/37 (78)	30/37 (81)

[a]The PRO questionnaire completion rate was calculated using the number of patients with non-missing PRO data at baseline and at a post-baseline visit, divided by the number of patients eligible for the respective visit. [b]For the global health status/QoL subscale. [c]For the FACT-G subscale. [d]For the VAS score. EORTC QLQ-C30, European Organisation for Research and Treatment of Cancer Quality of Life Questionnaire C30; FACT-M, Functional Assessment of Cancer Therapy-Melanoma; PRO, patient-reported outcome; VAS, visual analogue scale.

Supplemental Figure S1. PRESERV MEL study design. AE, adverse event; DMFS, distant metastasis-free survival; HCRU, healthcare resource utilization; HRQoL, health-related quality of life; OS, overall survival; RFS, relapse-free survival.

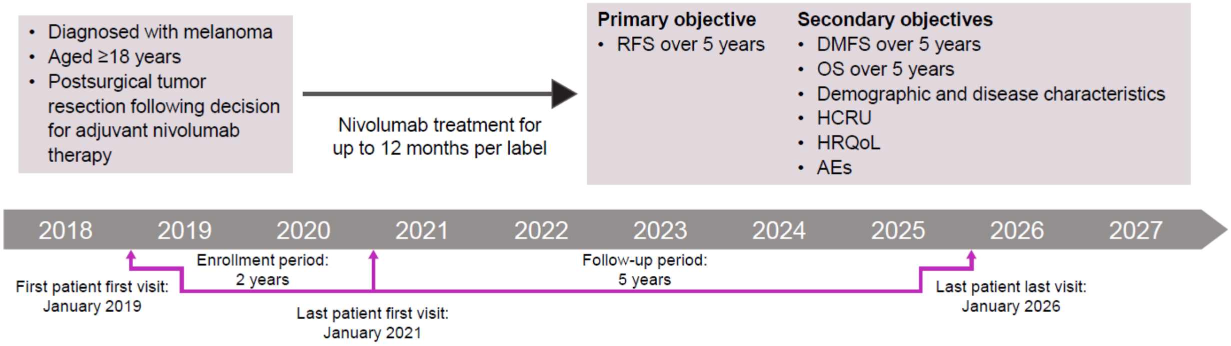

Supplement: Supplementary file 1 [file cancers-15-04823-s001.zip › cancers-2509599-supplementary.pdf]
